# Clinical study on multi-focused laser in the treatment of vulvar lichen sclerosus

**DOI:** 10.3389/fsurg.2022.919135

**Published:** 2022-09-15

**Authors:** Jing-Qiu Guo, Song-Yan Chen, Xue-Mei Chen, Jing-Quan Lu, Yu Song, He-Yu Liu, Li-Na Hu, Zheng-Yan Zhu

**Affiliations:** Department of Gynecology, Optics Valley Affiliated Tongren Hospital, Wuhan University, Wuhan, China

**Keywords:** vulvar lichen sclerosus, MF laser, radiofrequency, traditional Chinese medicine, clinical study

## Abstract

**Objective:**

To investigate the clinical effect of Multi-focused (MF) laser in the treatment of vulvar lichen sclerosus (VLS).

**Methods:**

In this single-center, randomized controlled trial, we compared the effect of fractionated MF laser with other treatments on patients with biopsy-proven VLS. Patients with VLS were enrolled in this study and randomly divided into three groups. Patients in the experimental group were treated with a CO_2_ laser, control group 1 was treated with radiofrequency, and control group 2 was treated topically with glucocorticoids and soaking with Chinese patent medicine. The pruritus degree, skin elasticity, skin color, lesion scope, and total score were compared before treatment, at one month after treatment, and three months after treatment.

**Results:**

One month after treatment, the pruritus degree, skin elasticity, skin color, lesion scope, and total score decreased in the experimental group, and the differences were statistically significant (*P* < 0.05). In control group 1, the differences in pruritus degree, skin color, and total score were statistically significant (*P* < 0.05), but the differences in skin elasticity and lesion scope were not statistically significant (*P* > 0.05). In control group 2, the differences in pruritus degree and total score were statistically significant (*P* < 0.05), but the differences in skin elasticity, skin color, and lesion scope were not statistically significant (*P* > 0.05). At one month after the end of treatment, the differences in pruritus degree, skin elasticity, skin color, lesion scope, and total score among the three groups were not statistically significant. At three months after the end of treatment, the differences in the scores of the five indicators were statistically significant.

**Conclusion:**

For the three treatment methods for VLS, topical corticosteroids + traditional Chinese medicine can quickly relieve itching symptoms in patients, but it cannot significantly improve skin elasticity, skin color, and lesion scope, and VLS easily relapses after treatment. Radiofrequency can improve itching symptoms and skin color but has poor effects on the change of skin elasticity and lesion scope. Multi-focused laser treatment can alleviate the degree of pruritus, improve skin color and elasticity, and narrow the lesion scope, and VLS will not relapse within three months after treatment.

## Introduction

Vulvar lichen sclerosus (VLS) is a chronic inflammatory non-neoplastic skin lesion of the vulva in women. The disease is characterized by atrophy and thinning of the skin and mucosa around the vulva and anus, with chronic progression and repeated attacks. Untimely and standardized treatment may lead to vulvar atrophy, adhesion, scar formation, and even loss of normal anatomy and function of the vulva, and the risk of local carcinogenesis will also increase. The disease is easily diagnosed and difficult to treat. Early diagnosis and intervention can improve the long-term prognosis of patients. However, at present, the understanding of VLS in domestic academic circles (including obstetrics, gynecology, and dermatology) is not unified. Approximately 90% of patients seek medical treatment for pruritus, while nearly 10% of asymptomatic patients are missed or misdiagnosed. There are significant regional differences in treatment plans. The effects of routine local applications of glucocorticoids are not lasting, and it is easy to relapse after drug withdrawal, while long-term use of hormones has obvious adverse reactions, and the overall curative effect is limited (1). MF laser is widely used in skin medical cosmetology. It can remove wrinkles, rejuvenate skin, and remove pigmented nevi, but it is rarely used to treat VLS (2).

## Subjects and methods

### Subjects

Patients with VLS treated at the Guanggu Gynecology Department of Wuhan Third Hospital from November 2020 to November 2021 were enrolled and randomly divided into three groups. In the experimental group, 25 patients with VLS were treated with an MF laser, control group 1 had 13 patients with VLS treated with radiofrequency, and control group 2 had 12 patients with VLS treated with 0.05% clobetasol propionate ointment combined with traditional Chinese medicine.

The inclusion criteria were as follows: (1) Pathologically diagnosed with VLS; (2) the ages ranged between 18 and 75 years old; (3) patients signed informed consent; (4) no other treatment regimen was used to treat VLS within three months. Patients who met the exclusion criteria were excluded from the study: (1) previously treated for intraepithelial neoplasia, carcinoma, or local radiotherapy; (2) with liver or kidney dysfunction; (3) with human immunodeficiency virus or syphilis infections, acute vaginitis, or cervical malignancy; (4) with cardiovascular disease; (5) with blood system diseases; (6) with hypertension or diabetes mellitus; (7) with mental illness; (8) pregnant or lactating women.

### Methods

Patients in the experimental group were treated with a CO_2_ laser, control group 1 was treated with radiofrequency, and control group 2 was treated with glucocorticoids and soaking with Chinese patent medicine.

The experimental group (laser treatment): The manufacturer of the laser therapeutic instrument is Wuhan Leijian Technology Co., Ltd., China, Ligenesis-MC30. Local anesthesia was given after disinfection. The patient was fixed in the lithotomy position. The energy density range was set within 40–50 mJ/pixel according to the location, color, and area of white spots. The front end of the laser hand tool was perpendicular to the white spot surface, and they were treated one by one. The number of image bundles was 81 dots according to the white spot area, peak power could be high, medium, and low, the pulse mode was set on “repeat,” the treatment time was 300–400 µs, and the distance between dots was 250–400 µm. The parameters were adjusted according to the patient's tolerance. Each area was treated with the laser 3–4 times. Local cold compresses were given within 12 h after treatment. The patient was forbidden to take a bath within 24 h or engage in vigorous exercise and hot water baths within 3–4 days, and sexual intercourse was prohibited within seven days after treatment. MF laser were performed once a month for 3 months by the same operator. Patients were administered with topical anesthetics before each MF laser treatment.

Control group 1 (radiofrequency treatment): With 5% 5-aminolevulinic acid (5-ALA) as a photosensitizer, a red light with a wavelength of 635 nm was used for radiofrequency. Each irradiation lasted for 10 min once a week for 10 weeks.

Control group 2: Clobetasol propionate ointment (0.05%) was used topically with traditional Chinese medicine soaking and washing for 3–4 months. Clobetasol propionate ointment was applied once a day for four weeks, then once every other day for four weeks, and, finally, twice a week for four weeks; the treatment course was three months. The dose referred to a fingertip unit (FTU); 1 FTU refers to the amount of medicine (about 0.5 g) extruded from the ointment tube with an opening of 5 mm that can cover the area from the fingertip of the index finger to the first interphalangeal joint. The traditional Chinese medicine consisted of *Fructus cnidii* and sweet wormwood. The combination of traditional Chinese medicine with Western medicine has been shown to enhance the curative effect, reduce the incidence of adverse reactions, and improve quality of life of patient with VLS (3).

### Observation indexes

The scores were recorded before treatment and at one and three months after treatment.

Observation indexes: (i) Clinical symptom evaluation: The curative effect indexes were evaluated according to the Cattaneo scoring system. Details are presented in Table 1.

**Table 1:** Cattaneo score.

Score	Pruritus degree	Skin elasticity	Skin color	Lesion scope
0	Non	Normal	Normal	0
1	Mild	Slightly poorer	Red	<30%
2	Moderate	Thin skin	Pink	30%–50%
3	Severe	Chapped skin	White	>50%

### Statistical data processing

Statistical analysis was performed using SPSS 20.0 software. Normally distributed measurement data were expressed as mean ± standard deviation (mean ± standard deviation), and non-normally distributed measurement data were expressed as median and interquartile ranges. Normally distributed data were evaluated using analysis of variance, and non-normally distributed data were evaluated using the Mann–Whitney U and Kruskal–Wallis nonparametric tests. All statistical tests were conducted using two-sided tests. *P* < 0.05 was considered statistically significant.

## Results

### Analysis of conditions among the three groups of patients before treatment

**Figure 1:**
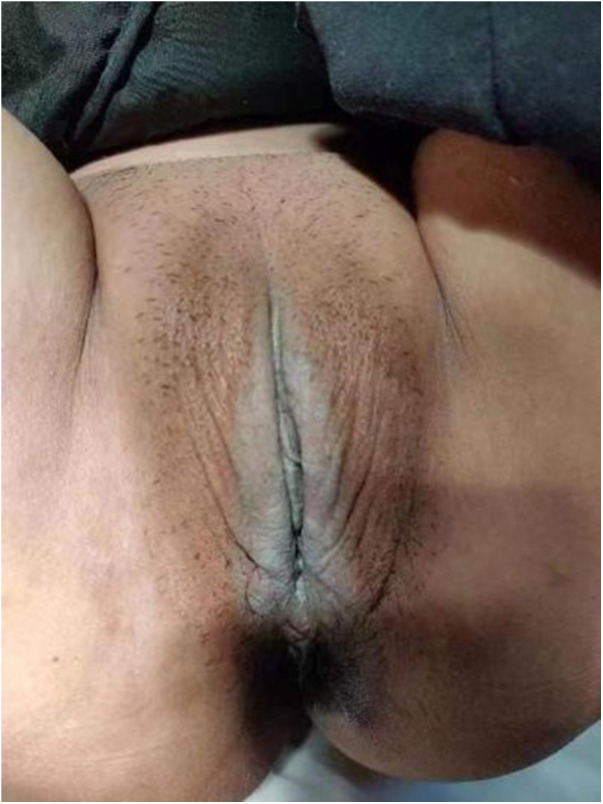
The genital area before MF laser treatment.

Before treatment, there were no significant differences among the three groups in the mean values of pruritus degree, skin elasticity and color, lesion scope, and total score (Table 2). Figure 1 shows the genital area of a patient with VLS before MF laser treatment.

**Table 2:** Comparison of three groups before treatment.

Scores before treatment	Experimental group	Control group 1	Control group 2	*P*
Pruritus degree	2.78 ± 0.422	2.73 ± 0.458	2.50 ± 0.522	0.221
Skin elasticity	1.35 ± 0.647	1.33 ± 0.617	1.33 ± 0.492	0.996
Skin color	2.17 ± 0.650	2.27 ± 0.594	2.33 ± 0.657	0.786
Lesion scope	2.39 ± 0.783	2.67 ± 0.617	2.00 ± 0.780	0.085
Total score	8.70 ± 1.329	9.00 ± 1.363	8.17 ± 1.239	0.221

### Analysis of conditions among the three groups of patients before and after treatment

The three groups of patients were self-controlled before and after treatment. In the experimental group, the degree of pruritus, skin elasticity and color, lesion scope, and total score decreased; the differences were statistically significant.

**Figure 2:**
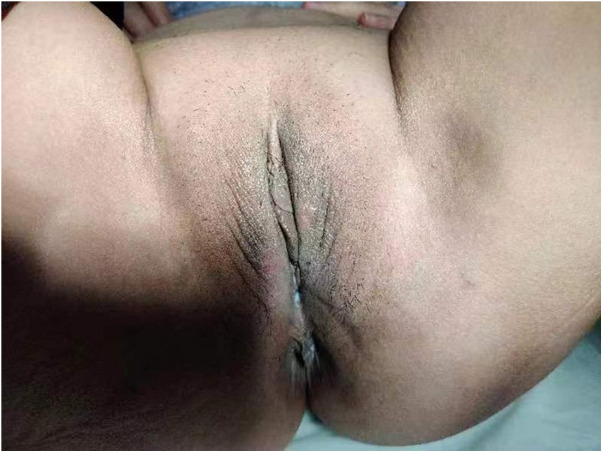
The genital area after MF laser treatment.

In control group 1, the degree of pruritus, skin color, and total score decreased, and the differences were all statistically significant. However, although skin elasticity and lesion scope decreased in control group 1, the differences were not statistically significant. Figure 2 shows the genital area of a patient with VLS after MF laser treatment.

In control group 2, the degree of pruritus and total score decreased, and the differences were statistically significant, but the differences in skin elasticity, color, and lesion scope were not statistically significant.

At three months after treatment, the pruritus degree was higher than one month after treatment, and the difference was statistically significant. At three months after treatment, although the pruritus score was lower than before treatment, the difference was not statistically significant (Table 3).

**Table 3:** Comparison of curative effect among three groups before and after treatment.

	Before treatment	One month after treatment	Three month after treatment	*P*
Pruritus degree in experimental group	2.78 ± 0.422	0.26 ± 0.449	0.17 ± 0.388	0.000
Skin elasticity in experimental group	1.35 ± 0.647	0.57 ± 0.662	0.57 ± 0.662	0.000
Skin color in experimental group	2.17 ± 0.650	1.13 ± 0.458	1.09 ± 0.417	0.000
Lesion scope in experimental group	2.39 ± 0.783	1.61 ± 0.656	1.30 ± 0.635	0.001
Total score in experimental group	8.70 ± 1.329	3.57 ± 1.199	3.13 ± 1.058	0.000
Pruritus degree in control group 1	2.73 ± 0.458	0.33 ± 0.488	0.33 ± 0.488	0.000
Skin elasticity in control group 1	1.33 ± 0.617	1.00 ± 0.535	0.93 ± 0.458	0.107
Skin color in control group 1	2.27 ± 0.594	1.33 ± 0.488	1.33 ± 0.488	0.000
Lesion scope in control group 1	2.67 ± 0.617	1.47 ± 0.516	1.47 ± 0.516	0.000
Total score in control group 1	9.00 ± 1.363	4.13 ± 0.915	4.07 ± 0.884	0.000
Pruritus degree in control group 2	2.50 ± 0.522	0.25 ± 0.452	1.50 ± 0.798	0.000
Skin elasticity in control group 2	1.33 ± 0.492	0.75 ± 0.866	1.08 ± 0.793	0.085
Skin color in control group 2	2.33 ± 0.657	1.75 ± 0.754	1.83 ± 0.718	0.136
Lesion scope in control group 2	2.00 ± 0.780	1.67 ± 0.492	1.75 ± 0.452	0.408
Total score in control group 2	8.17 ± 1.239	4.42 ± 1.311	5.58 ± 1.564	0.000

### Analysis of conditions among the three groups of patients one month after the End of treatment

One month after the end of treatment, there was a significant difference in skin color among the three groups. There was no significant difference in pruritus degree, skin elasticity, lesion scope, and total scores among the three groups (Table 4).

**Table 4:** Comparison among three groups at 1 month after treatment.

Scores at 1 month after treatment	Experimental group	Control group 1	Control group 2	*P*
Pruritus degree	0.26 ± 0.449	0.33 ± 0.488	0.25 ± 0.452	0.865
Skin elasticity	0.57 ± 0.662	1.00 ± 0.535	0.75 ± 0.866	0.122
Skin color	1.13 ± 0.458	1.33 ± 0.488	1.75 ± 0.754	0.025
Lesion scope	1.61 ± 0.656	1.47 ± 0.516	1.67 ± 0.492	0.643
Total score	3.57 ± 1.199	4.13 ± 0.915	4.42 ± 1.311	0.097

Note: Non parametric test was used for skin elasticity and skin color.

### Analysis of conditions among the three groups of patients three months after the end of treatment

At three months after the end of treatment, there were significant differences in pruritus degree, skin elasticity and color, and total scores among the three groups. However, there was no significant difference in lesion scope among the three groups (Table 5).

**Table 5:** Comparison among three groups at 3 months after treatment.

Scores at 3 months after treatment	Experimental group	Control group 1	Control group 2	*P*
Pruritus degree	0.17 ± 0.388	0.33 ± 0.488	1.50 ± 0.798	0.057
Skin elasticity	0.57 ± 0.662	0.93 ± 0.458	1.08 ± 0.793	0.002
Skin color	1.09 ± 0.417	1.33 ± 0.488	1.83 ± 0.718	0.003
Lesion scope	1.30 ± 0.635	1.47 ± 0.516	1.75 ± 0.452	0.095
Total score	3.13 ± 1.058	4.07 ± 0.884	5.58 ± 1.564	0.000

## Discussion

VLS is a chronic inflammatory skin disease. Local lymphocytic infiltration is often observed in skin biopsy pathology. About 90% of patients seek medical treatment due to symptoms. The most common symptom of VLS is intractable pruritus, usually at night. In severe cases, it can affect daily life and sleep. Other accompanying symptoms may include vulvar pain, dysuria, urinary pain, sexual dysfunction, and pain during sexual intercourse and defecation. Nearly 10% of VLS patients are completely asymptomatic and are usually found by chance or by doctors during a gynecological examination. The classic VLS skin texture changes are wrinkled or cellophane-like white patches, which can also be accompanied by irregular hyperkeratosis. The skin lesions mainly involve the labia majora and minora, prepuce of the clitoris, perineal body, and perianal skin, and are mostly symmetrically distributed. The hair growth area of the labia majora is usually not affected. The skin in the vulvar lesion area is fragile and primarily manifests as purpura, erosion, and chapping. If the lesion is not treated properly for a long time, it can cause the invagination of the vulva structure, the loss of the labia minora and the prepuce of the clitoris, or anterior-posterior combined adhesions, and finally, lead to the stenosis of the vaginal orifice and/or anus. Except for the natural remission of some VLS before puberty, most patients with VLS need active intervention and treatment. Furthermore, it is emphasized that even asymptomatic patients should be treated to delay the progression of the disease and improve the long-term prognosis. Currently in China, the local and external use of glucocorticoids is recommended as the first-line treatment of VLS (4–5). Physical therapy or surgical treatment can be chosen for patients with ineffective drug treatment or severe local adhesions. The purpose is to alleviate the itching symptoms, delay lesion progression, prevent complications, and improve the quality of life (6).

Literature reported that the incidence rates of VLS vary widely, from 1/70 to 1/1,000 (7). Since some patients are asymptomatic and do not see doctors in time, the actual incidence rates of VLS may be far underestimated. It is generally considered that there are two peaks in the age of onset of VLS. It usually occurs in postmenopausal women (average age 52.6 years old) and is not uncommon in preadolescent girls (average age 7.6 years old) (8, 9).

At present, the principle of MF lasers in the treatment of VLS is still unclear. The absorption of light energy by water molecules in the dermis achieves the ablation of the dermis and epidermis to reduce the occurrence of hyperkeratosis and lichen-like lesions to treat the disease (10). Therefore, MF laser treatment of VLS can not only improve the symptoms of vulvar pruritus but also improve the skin elasticity and color of lesions and reduce the lesion scope of patients. However, there are few reports on MF laser treatment of VLS. Pagano et al. (11) reported that an MF laser was used to remedy refractory VLS, but this literature failed to set up an effective control and lacked follow-up. No severe complications occurred during or after the treatment. Only a few patients reported red and swollen skin, and burn ointment gave immediate relief. These findings were consistent with the study by Di Donato et al. (12).

Radiofrequency is a type of high-frequency electromagnetic wave that has been widely used to treat tumors in the brain, liver, breast, thyroid, and other tissues in clinical practice. In recent years, its use in the of uterine leiomyomas of less than 5 cm has received satisfactory results (13). Electromagnetic waves act on the dermis and exert a thermal effect, resulting in vasodilation, accelerating blood flow, and increasing the permeability of the vascular endothelial cell membrane, improving the nutritional status of microvessels and nerve endings in the dermis so that the nerve terminals are no longer abnormally excited, and pruritus symptoms do not appear in the vulva. It also improves skin color and elasticity to a certain extent and reduces the lesion scope (14). Yang et al. reported on the therapeutic effect of radiofrequency in patients with VLS (15).

Topical glucocorticoids are currently the first-line treatment for VLS in China. Their local external application can effectively resist inflammation and allergies, promote capillary contraction, regulate immunity, inhibit mitosis, and improve vulvar symptoms with an acceptable short-term curative effect (16). However, its disadvantages are also obvious. The curative effect of this treatment is poor, and there is no significant improvement in skin elasticity, color, and lesion scope. It is easy to relapse after stopping treatment, long-term use brings noticeable adverse reactions to the skin, and the overall curative effect is general.

It is well-known that VLS is a disease that recurs easily. Patients with VLS should be given proper medical follow-up. In our study, the recurrence rate of patients treated with MF laser was lower than those treated with other three treatments. However, it should be noted that the period of this study was short, which is a limitation of the present study. In addition, only a small group of patients were recruited in the three groups. Future investigations with a larger sample size and a multi-center design are needed to validate the current findings.

## Conclusion

For these three treatment methods for VLS, topical corticosteroids + traditional Chinese medicine can quickly relieve itching symptoms in patients, but it cannot significantly improve skin elasticity, color, and lesion scope, and VLS easily relapses after treatment. Radiofrequency can improve itching symptoms and skin color but has poor effects on the change of skin elasticity and lesion scope. Multi-focused laser treatment can alleviate the degree of pruritus, improve skin color and elasticity, and narrow the lesion scope, and VLS will not relapse within three months after treatment.

## Data Availability

The original contributions presented in the study are included in the article/Supplementary Material, further inquiries can be directed to the corresponding author/s.
